# 

**Published:** 1886-11

**Authors:** 


### OUR ADVERTISERS.

SEE new advertisement of F. Stearns & Co., Detroit.

WM. R. WARNER & CO., PHILADELPHIA.—This is one of the oldest as well as one of the most reliable drug houses in America. They have an advertisement in this issue of THE JOURNAL, which will be found in the body of THE JOURNAL next to correspondence, to which we desire to direct the attention of our readers.

MAGNUS & HIGHTOWER, ATLANTA, GA.—We present in this issue of THE JOURNAL an advertisement of these gentlemen, to which the attention of physicians desiring to purchase instruments is invited. They have determined to go out of the instrument business and are offering their large stock at first cost. See their advertisement, next to correspondence, and write them for prices.

REED & CARNRICK, NEW YORK.—These gentlemen have a new advertisement in this issue of THE JOURNAL. They are the manufacturers of the justly celebrated "beef peptonoids" and peptonized cod-liver oil and milk, two preparations of much value. See their advertisement, and if you have not already tried them, write for samples and give them a trial.

STEPHENS' SADDLE-BAGS.—Our readers are requested to note the change in the advertisement of Messrs. Hopkins & Co., the manufacturers of these saddle-bags. We are confident that the saddle-bags and buggy-cases made by this firm are superior to any on the market. Mr. Phillips, the instrument dealer, in this city, informs us that they have superseded every other saddle-bag and buggy-case in this market, and that he is selling a large number of them.

